# Sarcopenia Predicts Cancer Mortality in Male but Not in Female Patients Undergoing Surgery for Cholangiocellular Carcinoma

**DOI:** 10.3390/cancers13215359

**Published:** 2021-10-26

**Authors:** Markus Sebastian Jördens, Lisa Heinrichs, Sven H. Loosen, Linda Wittig, Verena Keitel, David Schöler, Maximilian Schulze-Hagen, Christina Loberg, Gerald Antoch, Wolfram Trudo Knoefel, Tom Luedde, Georg Fluegen, Christoph Roderburg

**Affiliations:** 1 Department of Gastroenterology, Hepatology and Infectious Diseases, University Hospital Düsseldorf, Medical Faculty of Heinrich Heine University Düsseldorf, Moorenstr. 5, 40225 Düsseldorf, Germany; lisa.heinrichs@med.uni-duesseldorf.de (L.H.); Sven.Loosen@med.uni-duesseldorf.de (S.H.L.); linda.wittig@med.uni-duesseldorf.de (L.W.); Verena.Keitel@med.uni-duesseldorf.de (V.K.); david.schoeler@med.uni-duesseldorf.de (D.S.); luedde@hhu.de (T.L.); Christoph.Roderburg@med.uni-duesseldorf.de (C.R.); 2 Department for Diagnostic and Interventional Radiology, University Hospital Aachen, 52074 Aachen, Germany; mschulze@ukaachen.de; 3 Institute for Diagnostic and Interventional Radiology, University Hospital Düsseldorf, Medical Faculty of Heinrich Heine University Düsseldorf, 40225 Düsseldorf, Germany; christina.loberg@med.uni-duesseldorf.de (C.L.); gerald.antoch@med.uni-duesseldorf.de (G.A.); 4 Department of General, Visceral and Pediatric Surgery, University Hospital Düsseldorf, Medical Faculty of Heinrich Heine University Düsseldorf, 40225 Düsseldorf, Germany; knoefel@med.uni-duesseldorf.de (W.T.K.); Georg.Fluegen@med.uni-duesseldorf.de (G.F.)

**Keywords:** sarcopenia, cholangiocellular adenocarcinoma, CCA, surgery, prognostic marker

## Abstract

**Simple Summary:**

Cholangiocellular adenocarcinoma is the second most common primary liver tumor. If resectable, this is the therapeutic method of choice. Unfortunately, there are insufficient prognostic markers to determine which patients benefit most from surgery and which do not. The general condition of the patient, especially their muscle mass and quality, has become more and more the focus of recent research as a possible marker for therapeutic outcome. In our study we investigated how the preoperative muscle mass of patients with cholangiocellular adenocarcinoma affects postoperative overall survival. We showed that patients with a higher preoperative muscle mass had a significantly longer survival than patients with reduced muscle mass. Furthermore, we were able to show that this is particularly relevant in men and that in women preoperative muscle mass plays a significantly less important role in postoperative survival than in men.

**Abstract:**

Introduction: Surgery represents the only curative treatment option for patients with cholangiocarcinoma. However, complete tumor resection requires extensive surgery in many patients, and it is still debated which patients represent the ideal candidates for such therapy in terms of overall survival. Sarcopenia has been associated with an adverse outcome for various malignancies, but its role in the context of patients undergoing tumor resection for cholangiocellular adenocarcinoma (CCA) is only poorly understood. Here, we evaluated the role of sarcopenia in the outcome of CCA patients undergoing radical tumor resection. Methods: Pre-operative CT scans were used to assess the skeletal muscle index (L3SMI) as well as the psoas muscle index (L3PMI) in *n* = 76 patients receiving curative intended surgery for CCA. L3SMI and L3PMI were correlated with clinical and laboratory markers. Results: Patients with a skeletal muscle index or psoas muscle index above an established ideal cut-off (54.26 and 1.685 cm^2^/m^2^) showed a significantly better overall survival in Kaplan–Meier Curve analyses (L3SMI: 1814 days (95% CI: 520–3108) vs. 467 days (95% CI: 225–709) days; log rank X^2^(1) = 7.18, *p* = 0.007; L3PMI: 608 days (95% CI: 297–919) vs. 87 days (95% CI: 33–141), log rank X^2^(1) = 18.71; *p* < 0.001). Notably, these findings, especially for L3PMI, were confirmed in univariate (L3SMI: HR 0.962 (0.936–0.989); *p* = 0.006; L3PMI: HR 0.529 (0.366–0.766); *p* ≤ 0.001) and multivariate Cox regression analyses. Further analyses revealed that the prognostic value of both L3SMI and L3PMI was restricted to male patients, while in female patients survival was independent of the individual muscle mass. Conclusion: Measurement of muscle mass from preoperative CT scans represents an easily obtainable tool to estimate patient prognosis following curative surgery. The prognostic value was restricted to male patients, while in female patients these parameters did not reflect the patient outcome.

## 1. Introduction

Cholangiocellular carcinoma (CCA) is the second most common primary liver tumor with an incidence of 3/100,000 patients per year in Western countries [1]. In recent years, the incidence of CCA has constantly risen and today CCA represents a major global health burden. Despite numerous advances in molecular targeted therapies, radical tumor resection remains the only curative therapeutic option for patients with CCA. However, only about 20% of patients with CCA are amenable to curative surgical treatment, and around 60% of patients develop disease recurrence within 5 years [1]. The overall 5-year survival rate after surgical resection is 30%, compared to less than 1% for patients with advanced stage disease undergoing systemic chemotherapy. Complete tumor resection requires extensive surgery in many patients and is associated with substantial complications, which might even be life-threatening in severe cases [2,3,4]. Recently, several factors were suggested as prognostic and/or predictive markers to differentiate which patients benefit from surgical resection in terms of prolonged overall survival [5,6]. Nevertheless, these markers have yet to enter clinical routine, and easily accessible markers for the estimation of patient prognosis after surgery for CCA are urgently needed.

The Special Interest Group of the European Sarcopenia Working Group defined sarcopenia as “the progressive loss of muscle mass and strength with a risk of adverse outcomes such as disability, poor quality of life and death” [7,8]. In the last few years, sarcopenia has been identified as a potential factor when determining the outcome of patients with cancer, leading to cachexia, the common end point of deterioration of the general condition in tumor patients. Specifically, low skeletal muscle mass as well as low muscle quality were identified as negative prognostic factors in patients suffering from malignancies, including cholangiocellular carcinoma [9,10]. However, many of the available studies used unidimensional parameters to determine reduced muscle mass or muscle strength. In addition, potential influencing factors such as the patients’ sex were not taken into account. In this study, we measured the skeletal muscle index (L3SMI) and the psoas muscle index (L3PMI) to determine the sex-specific prognostic role of sarcopenia in patients undergoing surgery for CCA.

## 2. Methods

### 2.1. Selection of Study Patients

We used preoperative radiographic imaging (CT scan) and clinical follow-up data from 103 patients that underwent surgery for CCA in the Department of General, Visceral, and Pediatric Surgery at the University Hospital Düsseldorf between 2011 and 2021 (Detailed patients´ characteristics are presented in Table 1). Out of these, 25 patients were not resectable with curative intent (e.g., peritoneal metastases), and those were excluded from the analyses. Two patients were excluded for poor quality of the preoperative CT scan.

### 2.2. Analysis of Sarcopenia

**Figure 1:**
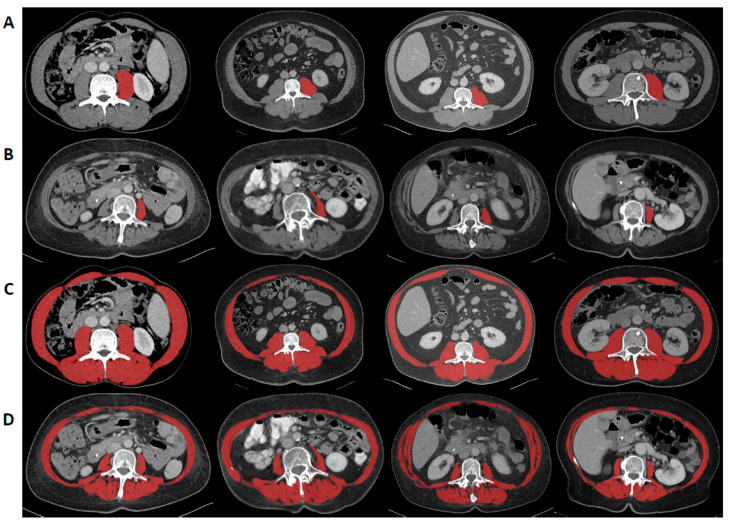
Assessment of the skeletal and the psoas muscle index at level of the third vertebra (L3SMI and L3PMI). L3SMI was assessed by measuring m. psoas, m. erector spinae, m. quadratus lumborum, m. rectus abdominis, m. transversus abdominis, m. obliquus abdominis internus, and m. obliquus abdominis externus in a single slice at the level of the third vertebra using the *3D Slicer* and normalizing it to the patient’s height. For L3PMI only the left m. psoas was measured and normalized to the patient’s body height. Examples for high (**A**,**C**) and low (**B**,**D**) L3PMI and L3SMI values.

CT scans with a slice thickness between 2–5 mm obtained during preoperative tumor staging were used to determine sarcopenia. To determine L3SMI, the areas of the m. psoas, m. erector spinae, m. quadratus lumborum, m. rectus abdominis, m. transversus abdominis, m. obliquus abdominis internus, and m. obliquus abdominis externus were measured in venous phase at the level of the third lumbar vertebra in a single slice using the *3D Slicer*, a semi-automatic segmentation tool, as recently described [11] (Figure 1). Using attenuation values between −29 and 150 Hounsfield Units (HU), muscles were quantified. The software automatically calculates the skeletal muscle area as an absolute value, which can be converted to the skeletal muscle index at the level of the third lumbar vertebra (L3SMI) by normalizing it to the patient’s height (cm^2^/m^2^). For the psoas muscle index at the level of the third lumbar vertebra (L3PMI), only the left m. psoas was measured using the *3D Slicer,* and the obtained value was normalized to the patient’s body height [9].

### 2.3. Statistical Analysis

Statistical analyses were performed as recently described in detail [9]. The Shapiro–Wilk test was used to determine normal distribution. Non-parametric data were compared using Mann–Whitney U test (two values) or Kruskal–Wallis test (more than two values). Spearman correlation was used for all correlation analyses. Box plots show medians, quartiles, and ranges. To determine the influence of various parameters on overall survival, Kaplan–Meier curves were generated, and the log-rank test was used to test statistical differences between groups. The optimal cut-off finder was used to determine optimal cut-off values for L3SMI and L3PMI [12]. Univariate and multivariate Cox regression analyses were performed to determine the prognostic value of individual variables with respect to overall survival. For this purpose, Hazard ratio (HR) and 95% confidence interval (CI 95%) are displayed. Statistical analyses were performed using SPSS 27 (SPSS, Chicago, IL, USA) unless otherwise stated. A *p*-value less than 0.05 is considered statistically significant.

## 3. Results

### 3.1. Baseline Characteristics of Patients

A total of 103 patients were included into this study. Out of these patients, 76 were included into the final analysis (Table 1). Of these, 51.3% were female, median age was 68.5 years (range 41–89). Median BMI was 26.08 kg/m^2^ (range 15.96–44.26). The localization of cholangiocellular carcinomas was as follows: intrahepatic CCA 68.4%, Klatskin 11.8%, distal CCA 3.9%, carcinoma of the gallbladder 15.8%. Out of this cohort, 9 patients developed postoperative biloma, 5 patients anastomotic insufficiency, and 10 patients impaired wound healing. Median overall survival was 431 days (Figure S1), recurrence-free survival at 12 months was 34.2%. Median preoperative L3SMI was 45.31 cm^2^/m^2^ (10.60–76.64), and the median preoperative L3PMI was 2.67 cm^2^/m^2^ (0.97–5.10) (see Table 1).

### 3.2. L3SMI Is Dependent on Sex, Age and Preoperative UICC Stage

**Figure 2:**
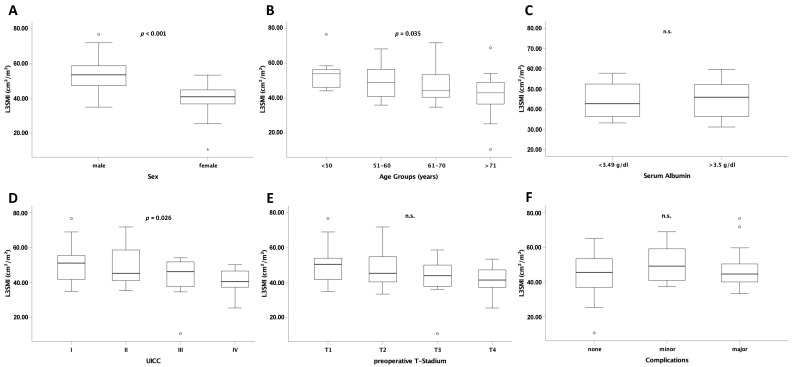
L3SMI is dependent on age, sex, and preoperative UICC stage. L3SMI was significantly different in male and female patients (**A**), different age groups (**B**), and UICC stage (**D**). For albumin serum concentration (**C**), T stage (**E**), and postoperative complications (**F**) no significantly different L3SMI levels were observed. Box plots show medians, quartiles, and ranges; ° = outlier; * = extreme value; n.s. = not significant.

L3SMI is defined as the total muscle area at the level of the third lumbar vertebra normalized to the individual patient´s height. To analyze sex or age-specific differences, we first compared L3SMI between the different sex and age groups. In these analyses, preoperative L3SMI was significantly higher in men than in women (*p* < 0.001; Figure 2A). Moreover, there was a statistically significant difference of higher L3SMI in younger patients (<50 years) than in patients >70 years (Figure 2B). Interestingly, there was no difference in L3SMI in patients with normal or reduced albumin serum concentrations as a marker for malnutrition (Figure 2C). In order to further evaluate clinical factors possibly affecting L3SMI we performed subgroup analyses and compared L3SMI among patients with different tumor stages. Notably, the local tumor stage (T) had no effect on median L3SMI while patients with an advanced overall tumor stage (according to UICC) displayed lower L3SMI than those in early stages (Figure 2D,E). Finally, median preoperative L3SMI had no effect on postoperative complications (Figure 2F).

### 3.3. L3PMI Is Dependent on Age, Sex, and Preoperative T- and UICC Stage

**Figure 3:**
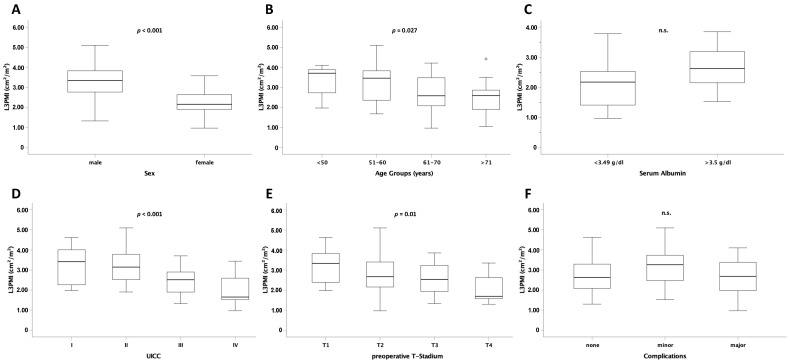
L3PMI is dependent on age, sex, and preoperative T and UICC stage. L3PMI was significantly different in male and female patients (**A**), different age groups (**B**), and UICC stage (**D**) as well as T stage (**E**). For albumin serum concentration (**C**) and postoperative complications (**F**) no significantly different L3PMI levels were observed. Box plots show medians, quartiles, and ranges; ° = outlier; n.s. = not significant.

Similar to L3SMI, the L3 psoas muscle index (L3PMI) was significantly different between the different sex and age groups (Figure 3A,B). Furthermore, median L3PMI was significantly higher in patients with early overall tumor stage (according to UICC) or local tumor stage (T) than in patients with advanced UICC or T stage (*p* < 0.001 and *p* = 0.010) (Figure 3D,E). As shown for L3SMI, L3PMI was not different depending on albumin serum concentration (Figure 3C). Moreover, the preoperative median value did not affect postoperative complications (Figure 3F).

### 3.4. L3SMI Is a Predictor for Postoperative Survival

**Figure 4:**
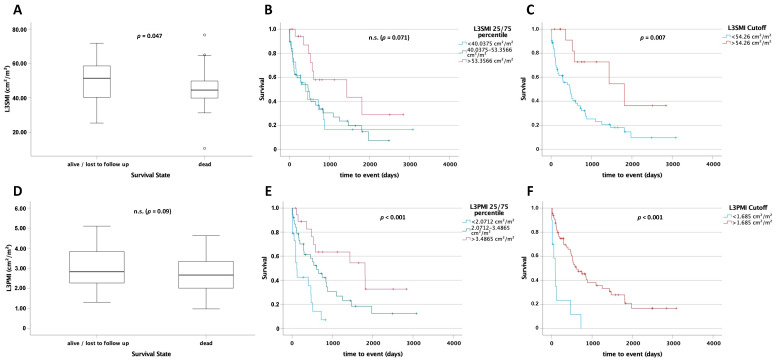
L3SMI and L3PMI are predictors for postoperative survival. L3SMI (**A**) and L3PMI (**D**) levels in survivors were higher than those in deceased patients. Patients with an L3SMI (**B**) or L3PMI (**E**) above the 75th percentile had a significantly higher overall survival. Using an optimal cut-off of 54.26 cm^2^/m^2^ for L3SMI (**C**) and 1.685 cm^2^/m^2^ for L3PMI (**F**), patients above these values had a significantly higher overall survival. ° = outlier; n.s. = not significant.

Based on the previously described prognostic role of sarcopenia and cachexia in patients with cancer we next compared L3SMI values between patients who died during long-term follow-up and long-term survivors. Survivors had a significantly higher L3SMI (*p* = 0.047; Figure 4A). To further analyze the prognostic role of L3SMI in patients with CCA we next performed Kaplan–Meier analysis. Notably, patients with a preoperative L3SMI above the 75th percentile (>40.0375 cm^2^/m^2^) of all patients showed a clear trend toward improved postoperative overall survival, when compared to the other patients (1435 (95% CI: 0–3222) days vs. 473 (95% CI: 218–728) days and 403 (95% CI: 109–697) days; log rank X^2^(2) = 5.303, *p* = 0.071; Figure 4B). We next calculated an optimal cut-off to best stratify patients according to their postoperative prognosis using the *optimal cut-off finder* [12]. Patients with an L3SMI above 54.26 cm^2^/m^2^ had a significantly improved postoperative overall survival, compared to patients with an L3SMI below this value (1814 days (95% CI: 520–3108) vs. 467 days (95% CI: 225–709) days; log rank X^2^(1) = 7.18 *p* = 0.007; Figure 4C).

### 3.5. L3PMI Is a Predictor for Postoperative Survival

As with L3SMI we compared L3PMI between survivors and non-survivors. A statistically non-significant trend for a higher psoas muscle index (L3PMI) was identified in patients who displayed long-term survival compared to deceased patients (*p* = 0.09, Figure 4D). Moreover, patients with a value above the 75th percentile had a significantly improved overall survival compared to patients between the 25th and 75th and below the 25th percentile (1810 days (95% CI: 64–3556) vs. 608 days (95% CI: 304–912) vs. 118 days (95% CI: 70–166); log rank X^2^(2) = 18.95; *p* < 0.001; Figure 4E). Using the *optimal cut-off finder* [12] for L3PMI, a cut-off value of 1.685 cm^2^/m^2^ was identified. Again, overall survival was significantly higher in patients with an L3PMI greater than 1.685 cm^2^/m^2^ (657 days (95% CI: 320–994) vs. 87 days (95% CI: 81–93), log rank X^2^(1) = 22.02; *p* < 0.001; Figure 4F).

### 3.6. L3PMI Is an Independent Prognostic Factor for Postoperative Overall Survival

To further support the prognostic value of L3SMI and L3PMI, we performed univariate and multivariate Cox regression analyses including various disease and patients´ related characteristics (Table 2). In these analyses, both L3SMI and L3PMI were identified as prognostic factors for postoperative overall survival in the univariate analysis (HR 0.962 (0.936–0.989); *p* = 0.006 and HR 0.529 (0.366–0.766); *p* < 0.001)). Furthermore, the relevance of L3PMI was independent of potentially confounding variables including age, tumor markers, and other laboratory values associated with organ dysfunction (HR 0.285 (0.098–0.831); *p* = 0.022). To further investigate the predictive power of L3SMI and L3PMI we performed ROC analyses. Here, we examined L3SMI and L3PMI with respect to 3-, 6-, 12-month and overall survival and compared the obtained AUC values with the AUC values of age and UICC stage as other relevant prognostic markers (Table 2) and L3SMI- and L3PMI-influencing (Figure 2B,D and Figure 3B,D) markers (Table S1). For overall survival, the values for both L3SMI (AUC 0.64) and L3PMI (AUC 0.62) were similar to those for UICC stage (AUC 0.62) and patient age (AUC 0.62). For 3- and 6-month survival, especially high AUC values for L3PMI (3 months: 0.73, 6 months 0.73) were recorded, further underlining the prognostic value of this marker.

### 3.7. L3SMI and L3PMI Correlate Negatively with Markers of Inflammation

To further illuminate the underlying mechanisms for the prognostic relevance of L3SMI and L3PMI, we performed correlation analyses with several clinical and laboratory markers. In addition to positive correlations of L3SMI and L3PMI with height, body weight, and BMI, strong negative correlations with the tumor marker CA19-9, the inflammatory marker CRP, and the leukocyte count were particularly striking (Table 3). 

### 3.8. L3SMI and L3PMI Are Sex-Specific Predictors for Postoperative Overall Survival

**Figure 5:**
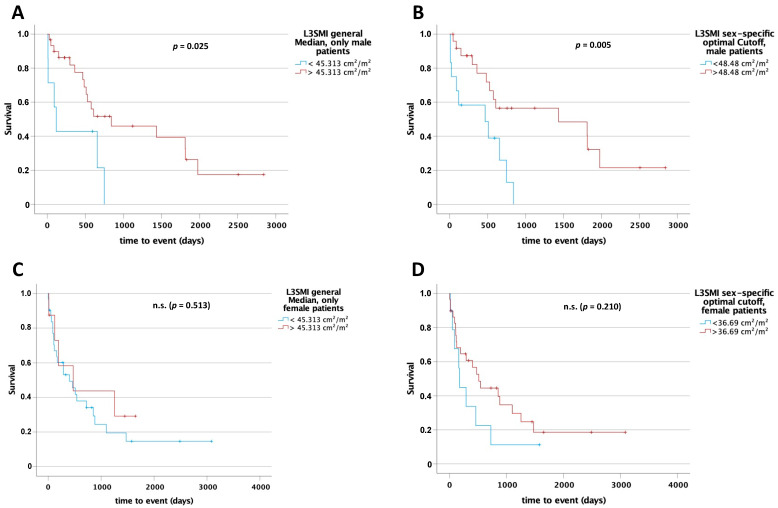
L3SMI is a sex-specific preoperative predictor for overall survival in patients undergoing surgery for CCA. Using the median (45.313 cm^2^/m^2^) for L3SMI of the total study cohort as a cut-off for overall survival in male (**A**) and female (**C**) subgroups, only in male patients could a significant difference in overall survival be identified. After using sex-specific cut-off values differences were seen in male patients (48.48 cm^2^/m^2^) only (**B**,**D**). n.s. = not significant.

**Figure 6:**
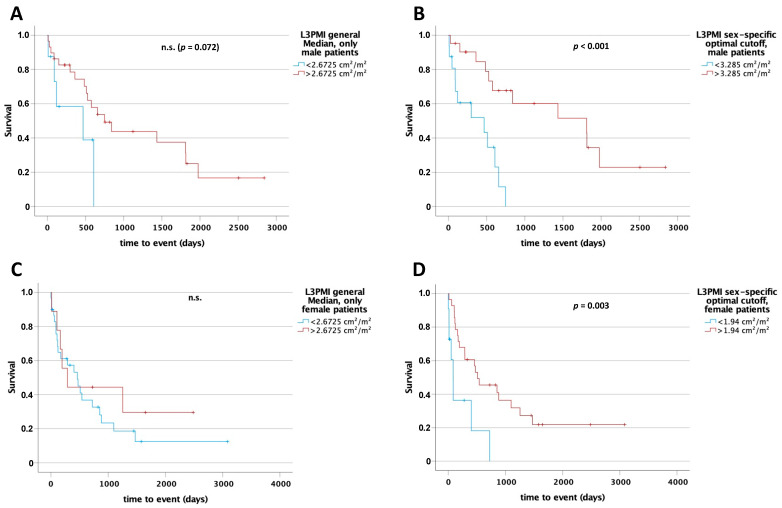
L3PMI is a sex-specific preoperative predictor for overall survival in patients undergoing surgery for CCA. Using the median (2.6725 cm^2^/m^2^) for L3PMI of the total study cohort as a cut-off for overall survival in male (**A**) and female (**C**) subgroups, only in male patients could a clear trend for improved overall survival be identified. After using sex-specific cut-off values were statistically significant differences seen in both male and female patients (**B**,**D**). n.s. = not significant.

Since muscle mass and quality tends to differ between male and female, we hypothesized that the predictive values of L3SMI and L3PMI might be sex-specific. Therefore, we first compared overall survival between men and women in our cohort that underwent surgery in curative intention. However, both displayed an almost identical survival post-surgery (*p* = 0.264; Figure S2). To compare the prognostic value of L3SMI and L3PMI, we performed Kaplan–Meier analyses separately in male and female patients, using the median for L3SMI and L3PMI in the whole cohort as cut-off. Interestingly, for L3SMI a significant difference regarding survival between patients above and below the median was only apparent in the male cohort (L3SMI 45.313 cm^2^/m^2^: 841 days [95% CI: 0–1912] vs. 118 days (95% CI: 46–190), log rank X^2^(1) = 5.042; *p* = 0.025 and L3PMI 2.6725 cm^2^/m^2^: 749 days (95% CI: 376–1122) vs. 467 days (95% CI: 0–1175), log rank X^2^(1) = 3.24; *p* = 0.072; Figure 5A and Figure 6A). The *optimal cut-off finder* [12] was used to determine a sex-specific cut-off value to differentiate between patients with long or short postoperative survival. In the male cohort, a statistically significant difference in overall survival was identified between patients above and below the cut-off (L3SMI 48.48 cm^2^/m^2^: 1435 days (95% CI: 0–3195) vs. 467 days (95% CI: 0–1054), log rank X^2^(1) = 7.73; *p* = 0.005 and L3PMI 3.285 cm^2^/m^2^: 1810 days (95% CI: 330–3290) vs. 467 days (95% CI: 0–1008), log rank X^2^(1) = 12.482; *p* < 0.001; Figure 5B and Figure 6B). In the female cohort, there was only a statistically significant difference using the sex-specific optimal cut-off value for L3PMI of 1.94 cm^2^/m^2^ (512 days (95% CI: 49–975) vs. 86 days (95% CI: 38–134), log rank X^2^(1) = 8.97; *p* = 0.003; Figure 5C,D and Figure 6C,D). For L3SMI, there was no statistically significant difference in overall survival for female patients despite an optimal sex-specific cut-off (512 days (95% CI: 294–730) vs. 172 days (95% CI: 146–198), log rank X^2^(1) = 1.574; *p* = 0.210). Finally, univariate Cox regression analysis performed separately for males and females confirmed the prognostic role of L3SMI and, in particular, L3PMI only in men, while in females both parameters did not reflect patient survival (Table 4). In summary, our data argue for a previously unrecognized role of L3SMI and L3PMI as sex-specific, easily accessible prognostic markers in CCA.

## 4. Discussion

Selection of patients appropriate for curative surgery for cholangiocellular adenocarcinoma is challenging. Today, no reliable prognostic markers have been established to determine which patients will benefit from tumor resection in the long term. Here, we demonstrated that measurement of muscle mass from preoperative CT scans represents an easily obtainable tool to estimate patients´ prognoses following curative surgery. Strikingly, the prognostic value of muscle mass was restricted to male patients, while in female patients, this parameter did not reflect the patients´ outcomes.

A progressive loss of muscle strength and muscle mass—referred to as sarcopenia—is part of the normal aging process. Sarcopenia has a higher prevalence in elderly patients, increasing from the third decade to the sixth decade and reaching a constant level [13]. Furthermore, apart from a general increase at a higher age independent of sex, it is even more frequent in older women than men and is connected with increasing functional impairment [13]. In the context of numerous diseases, the process of muscle loss and degeneration can be accentuated and accelerated. Cachexia represents the endpoint of this process and is characterized by the parallel occurrence of muscle loss and inflammatory processes [14,15]. While sarcopenia can at least be slowed down by appropriate therapies, cachexia is irreversible in many patients and has a life-shortening effect. We demonstrated that cachexia occurs frequently in patients with cholangiocarcinoma and showed that overall survival is impaired in such patients. In our cohort of patients, sarcopenia according to an impaired L3SMI and/or L3PMI was more pronounced in patients with advanced tumor stages. Notably, our findings on a prognostic role of L3SMI/L3PMI in cholangiocellular carcinoma are consistent with other studies on sarcopenia in various tumor or non-tumor diseases [10,16], supporting the role of sarcopenia as a prognostic marker in cancer. Methods such as bioelectrical impedance analysis or ultrasound can be used to measure body composition [17,18]. Yet, since preoperative imaging is available for all patients with cancer and can be evaluated according to the methodology described, we decided to use L3SMI and L3PMI as markers for muscle mass and sarcopenia. The advantage of the method chosen here is the ease of availability. In particular, L3PMI is a very easily and reliably determinable parameter that proved to be a robust predictor of oncological endpoints in our analyses. The use of L3SMI/L3PMI in clinical routine was supported by our results, demonstrating that these parameters are independent prognostic markers for postoperative overall survival in patients undergoing surgery for CCA. Strikingly, L3SMI and L3PMI were even independent from serum albumin concentration, which is an established marker of the nutrition state, highlighting that the effect of muscle loss on the patient´s survival is complex and goes far beyond simple malnutrition.

Physical inactivity, often increasing with older age, but also seen in cancer patients, may be partly responsible for sarcopenic changes. In particular, the type I muscle fibers predominant in postural muscles appear to be susceptible to inactivity [19]. Furthermore, sarcopenia is also influenced by the loss of motor units in the aging nervous system [20,21]. We demonstrated that L3SMI and L3PMI reflect the prognosis in male but not in female patients undergoing surgery for CCA. In this respect, our analyses add to the current body of work focused on the impact of sarcopenia on cancer by focusing on gender-specific differences. In this regard, our work is in line with previous findings on the lack of association between sarcopenia and post-transplant mortality in females [22]. Interestingly, the authors were able to generate a significant cut-off value of 48cm^2^/m^2^ for L3SMI for the prediction of post-transplant survival only for men, which is almost identical to our optimal sex-specific cut-off value for men of 48.48 cm^2^/m^2^. Analogous to our work, no optimal cut-off value for L3SMI in women could be identified. From a pathophysiological point of view our data might be explained by the effect of specific hormonal changes, such as a decreased testosterone levels, which might have an impact on muscle mass and strength in males [23,24]. In addition, dehydroepiandrosterone (DHEA) has been shown to have an inhibitory effect on IL-6 production [25]. Furthermore, a strong estrogenic influence on different mechanisms affecting skeletal muscle protein turnover regulation, including the IGF-1/Akt/mTORC1 cascade, the Ubiquitin-proteasome system, and the autophagy lysosome system, are suspected. Changes in estrogen serum concentrations thus have the potential to alter skeletal muscle metabolism [26].

High IL-6 concentrations could be associated with increasing muscle weakness and immobility, especially in elderly patients [27,28,29]. In addition, IL-6 seems to contribute to loss of appetite in anorexia [30]. However, IL-6 serum levels are elevated in various tumor diseases and associated with aggressive tumor growth, impaired response to therapy, and poor prognosis [31,32,33,34]. While our cohort did not feature IL-6 concentrations, we have an almost complete set of data regarding CRP and leukocyte counts as markers of systemic inflammatory reaction. Strikingly, both CRP and leukocytes were correlated with L3SMI and L3PMI, providing another explanation of how inflammation impacts the prognosis of cancer patients (Table 4).

Overall, our study is subject to some limitations. First, the data examined were collected retrospectively and therefore not specifically for our research question. Furthermore, our cohort of 76 patients is respectable for a rare disease but remains small for gender-specific subgroup analyses. Finally, our study includes patients from a single center only. While this bears some advantages concerning standardization of patients’ treatment and data collection, we cannot fully rule out that inclusion of patients from different centers (e.g., non-tertiary care clinics) might have changed some of the findings. We are aware that several options exist for determining L3SMI. In order to do justice to this fact and to create as much transparency as possible, we performed all analyses with both the unit cm^2^/m^2^ and the unit mm^2^/cm (Figures S3–S7). Both analyses yielded almost identical results, which further strengthened the validity of our analyses. Taking note of these limitations, our study is the first to demonstrate a gender-specific role of sarcopenia as a prognostic marker in CCA. Our results should trigger further research in order to establish muscle mass as a prognostic and/or predictive marker in clinical routine. As such, we propose additional studies using larger and multicenter cohorts to confirm our results. In this context, interventional study designs to test whether treatment of muscle loss affects the patients´ overall survival bear the potential to fundamentally change the management of patients with CCA. In detail, our data argue for clinical trials testing a potential beneficial effect of preoperative nutrition and training strategies in the context of CCA surgery. Given the controversial findings in males and females, our study clearly highlights the importance of gender stratification in analyses and studies addressing sarcopenia in cancer.

## 5. Conclusions

We identified L3SMI and L3PMI as novel prognostic markers for overall survival after surgery for cholangiocellular adenocarcinoma. From both a clinical and a pathophysiological point of view, our data on the gender-specific function of these parameters add elegantly to the existing literature on the role of sarcopenia in cancer and support the use of L3SMI/L3PMI in the preoperative assessment of patients with CCA.

## Figures and Tables

**Table 1:** Study population.

Parameter	Study Cohort
CCA patients	*n* = 76
Sex (%)	
Male	48.7 (37)
Female	51.3 (39)
Age (years, median and range)	68.5 (41–89)
BMI (kg/m^2^, median and range)	26.08 (15.96–44.26)
Tumor localization (%)	
iCCA	68.4 (52)
Klatskin	11.8 (9)
dCCA	3.9 (3)
gallbladder	15.8 (12)
Staging (%)	
UICC I	26.3 (20)
UICC II	28.9 (22)
UICC III	23.7 (18)
UICC IV	18.4 (14)
Disease control at 12 months	34.2
Postoperative complications	
None	39.5 (30)
Minor	21.1 (16)
Serious	39.5 (30)
Overall survival (days, median and range)	431 (2–3087)
Recurrence-free survival at 12 months (%)	34.2% (26)
L3SMI (cm^2^/m^2^, median and range)	45.31 (10.60–76.64)
L3PMI (cm^2^/m^2^, median and range)	2.67 (0.97–5.10)

CCA: Cholangiocellular adenocarcinoma, iCCA: Intrahepatic cholangiocellular adenocarcinoma, dCCA: Distal cholangiocellular adenocarcinoma, UICC: Union for International Cancer Control, L3SMI: L3 skeletal muscle index, L3PMI: L3 psoas muscle index.

**Table 2:** Univariate and multivariate Cox regression analysis for the prediction of postoperative overall survival.

Parameter	Univariate Cox Regression		Multivariate Cox Regression	
	*p*-Value	Hazard Ratio (95% CI)	*p*-Value	Hazard Ratio (95% CI)
Sex	0.266	0.730 (0.419–1.271)		
Age	0.027	1.034 (1.004–1.064)	0.743	1.018 (0.917–1.129)
Height cm	0.855	0,997 (0.971–1.025)		
Weight kg	0.213	0.990 (0.976–1.006)		
BMI	0.208	0.963 (0.909–1.021)		
Sodium	0.684	0.986 (0.922–1.054)		
Potassium	0.149	0.647 (0.358–1.169)	0.327	0.551 (0.167–1.818)
Creatinine	0.407	0.787 (0.446–1.388)		
GFR mL/min	0.484	0.996 (0.985–1.007)		
Urea	0.710	1.004 (0.981–1.028)		
Uric acid	0.920	1.002 (0.960–1.046)		
Bilirubin	0.191	1.116 (0.947–1.316)		
AST	0.014	1.000 (1.000–1.001)	0.879	0.999 (0.984–1.014)
γGT	0.297	1.000 (1.000–1.001)		
AP	0.195	1.001 (1.000–1.002)		
Albumin	0.569	0.803 (0.377–1.710)		
TSH	0.885	0.986 (0.811–1.198)		
CEA	0.112	1.014 (0.997–1.031)	0.281	1.013 (0.989–1.037)
AFP	0.217	1.017 (0.990–1.044)		
CA19-9	0.069	1.000 (1.000–1.000)	0.967	1.000 (1.000–1.000)
INR	0.130	1.334 (0.919–1.937)	0.604	4.966 (0.012–2127.682)
aPTT	0.160	1.032 (0.988–1.078)		
UICC stage	<0.001			
L3SMI (cm^2^/m^2^)	0.006	0.962 (0.936–0.989)	0.838	1.012 (0.905–1.132)
L3PMI (cm^2^/m^2^)	<0.001	0.529 (0.366–0.766)	0.022	0.285 (0.098–0.831)

BMI: Body-Mass Index, GFR: glomerular filtration rate, AST: aspartate-aminotransferase, γGT: γ-glutamyltransferase; AP: Alkaline phosphatase; TSH: Thyroid-stimulating hormone, CEA: Carcinoembryonic antigen, AFP: α-fetoprotein, CA19-9: Carbohydrate antigen 19-9, INR: International normalized ratio, aPTT: activated partial thromboplastin time; for multivariate Cox regression analysis variables with a *p*-value < 0.150 in univariate Cox regression analysis were included.

**Table 3:** Spearman Correlation of L3SMI and L3PMI with relevant parameters.

Parameter	L3SMI		L3PMI	
	Correlation Coefficient	Significance (Two-Sided)	Correlation Coefficient	Significance (Two-Sided)
Age	−0.370	0.001	−0.393	<0.001
Height cm	0.330	0.004	0.252	0.028
Weight kg	0.649	<0.001	0.460	<0.001
BMI	0.586	<0.001	0.411	<0.001
Sodium	0.114	0.335	0.165	0.160
Potassium	−0.055	0.641	−0.129	0.274
Creatinine	0.236	0.042	0.085	0.468
GFR mL/min	0.193	0.104	0.334	0.004
Urea	−0.002	0.988	−0.022	0.852
Uric acid	0.293	0.103	0.126	0.493
Bilirubin	−0.193	0.109	−0.052	0.668
AST	−0.171	0.145	−0.099	0.403
γGT	−0.072	0.551	−0.095	0.434
AP	−0.428	0.001	−0.383	0.003
Albumin	0.118	0.573	0.268	0.196
TSH	−0.037	0.782	−0.147	0.274
CEA	−0.184	0.268	−0.253	0.125
AFP	−0.224	0.183	−0.054	0.751
CA19-9	−0.395	0.008	−0.545	<0.001
INR	−0.085	0.511	−0.070	0.589
aPTT	−0.230	0.072	−0.124	0.336
CRP	−0.313	0.007	−0.388	0.001
Leucocytes	−0.570	0.002	−0.396	<0.001

BMI: Body-Mass-Index, GFR: glomerular filtration rate, AST: aspartate-aminotransferase, γGT: γ-glutamyltransferase; AP: Alkaline phosphatase; TSH: Thyroid-stimulating hormone, CEA: Carcinoembryonic antigen, AFP: α-fetoprotein, CA19-9: Carbohydrate antigen 19-9, INR: International.

**Table 4:** Univariate Cox regression analysis for the prediction of sex-specific postoperative overall survival.

Parameter	Sex	Univariate Cox Regression	
		*p*-Value	Hazard Ratio (95% CI)
Age	m	0.288	1.023 (0.981–1.068)
	f	0.087	1.035 (0.995–1.078)
Height cm	m	0.673	0.989 (0.937–1.043)
	f	0.172	1.035 (0.985–1.088)
Weight kg	m	0.094	0.977 (0.951–1.004)
	f	0.384	1.013 (0.984–1.043)
BMI	m	0.132	0.932 (0.851–1.021)
	f	0.683	1.019 (0.930–1.116)
Sodium	m	0.059	0.887 (0.783–1.005)
	f	0.381	1.042 (0.950–1.144)
Potassium	m	0.156	0.477 (0.172–1.326)
	f	0.360	0.716 (0.349–1.466)
Creatinine	m	0.420	1.877 (0.407–8.660)
	f	0.324	0.720 (0.375–1.384)
GFR	m	0.174	0.986 (0.965–1.006)
	f	0.993	1.000 (0.987–1.013)
Urea	m	0.118	1.037 (0.991–1.085)
	f	0.816	0.997 (0.971–1.023)
Uric acid	f	0.538	0.772 (0.338–1.761)
	f	0.856	1.004 (0.963–1.047)
Bilirubin	m	0.002	3.925 (1.676–9.190)
	f	0.975	0.997 (0.810–1.227)
AST	m	0.879	1.001 (0.993–1.008)
	f	0.028	1.000 (1.000–1.001)
γGT	m	0.135	1.003 (0.999–1.006)
	f	0.604	1.000 (0.999–1.001)
AP	m	0.003	1.011 (1.004–1.018)
	f	0.721	1.000 (0.999–1.002)
Albumin	m	0.133	0.153 (0.013–1.774)
	f	0.836	0.916 (0.399–2.102)
TSH	m	0.618	0.904 (0.608–1.345)
	f	0.899	0.984 (0.770–1.257)
CEA	m	0.262	1.048 (0.965–1.139)
	f	0.235	1.011 (0.993–1.029)
AFP	m	0.342	1.021 (0.978–1.065)
	f	0.131	1.027 (0.992–1.064)
CA19-9	m	0.061	1.000 (1.000–1.000)
	f	0.958	1.000 (1.000–1.000)
INR	m	0.699	2.320 (0.033–165.530)
	f	0.347	3.153 (0.287–34.599)
aPTT	m	0.341	0.931 (0.802–1.079)
	f	0.115	1.035 (0.992–1.081)
UICC stage	m	0.040	
	f	0.004	
L3SMI (cm^2^/m^2^)	m	0.018	0.940 (0.893–0.990)
	f	0.283	0.967 (0.921–1.015)
L3PMI (cm^2^/m^2^)	m	0.001	0.393 (0.224–0.688)
	f	0.069	0.468 (0.206–1.060)

BMI: Body-Mass Index, GFR: glomerular filtration rate, AST: aspartate-aminotransferase, γGT: γ-glutamyltransferase; AP: Alkaline phosphatase; TSH: Thyroid-stimulating hormone, CEA: Carcinoembryonic antigen, AFP: α-fetoprotein, CA19-9: Carbohydrate antigen 19-9, INR: International normalized ratio, aPTT: activated partial thromboplastin time, m: male, f: female.

## Data Availability

Data are available upon request from the Department of Gastroenterology, Hepatology and Infectious Diseases of the University Hospital Düsseldorf for researchers who meet the criteria for access to confidential data: Wissenschaft.Gastro@med.uni-duesseldorf.de.

## Supplementary Materials

The following are available online at https://www.mdpi.com/article/10.3390/cancers13215359/s1, Figure S1: Kaplan–Meier analysis of overall survival in our cohort, Figure S2: Kaplan–Meier analysis of overall survival regarding sex differences, Figures S3–S7: Representation of Figure 2, Figure 3, Figure 4, Figure 5 and Figure 6 in mm^2^/cm, Table S1: ROC-Analysis of L3SMI, L3PMI, UICC stage and age.
