# Supplementary material for: Sarcopenia Predicts Cancer Mortality in Male but Not in Female Patients Undergoing Surgery for Cholangiocellular Carcinoma
Source: Cancers (Basel). 2021 Oct 26;13(21):5359. doi: 10.3390/cancers13215359 (PMC8582463; doi:10.3390/cancers13215359)
Supplement: Supplementary file 1 [file cancers-13-05359-s001.zip › cancers-1411052-supplementary.pdf]

cancers

Supplementary Materials

Sarcopenia Predicts Cancer Mortality in Male but Not in Female Patients Undergoing Surgery for Cholangiocellular Carcinoma

Markus Sebastian Jördens, Lisa Heinrichs, Sven H. Loosen, Linda Wittig, Verena Keitel, David Schöler, Maximilian Schulze-Hagen, Christina Loberg, Gerald Antoch, Wolfram Trudo Knoefel, Tom Luedde, Georg Fluegen and Christoph Roderburg

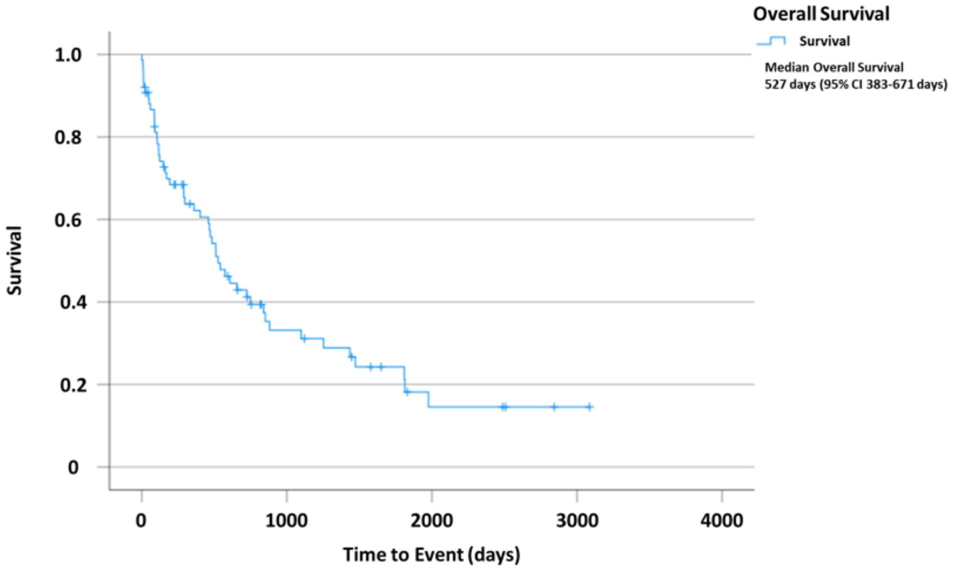

Figure S1. Kaplan–Meier analysis of overall survival in our cohort.

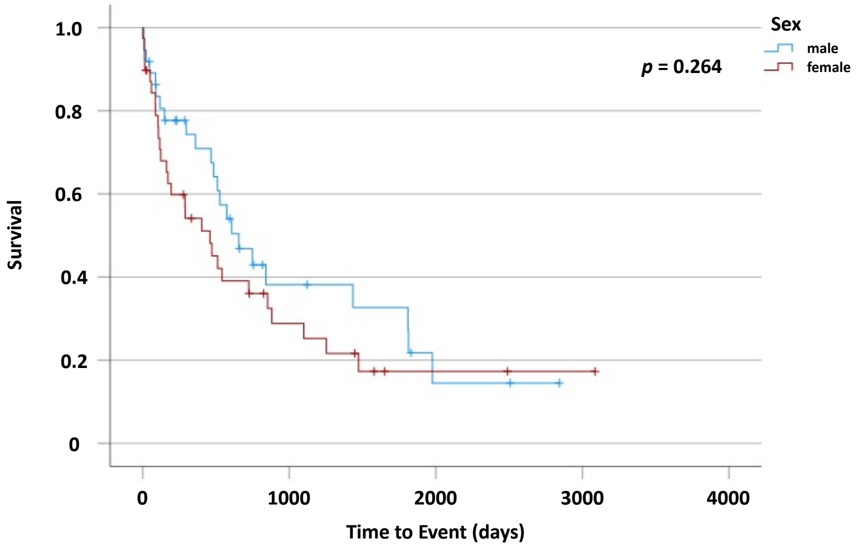

Figure S2. Kaplan–Meier analysis of overall survival regarding sex differences.

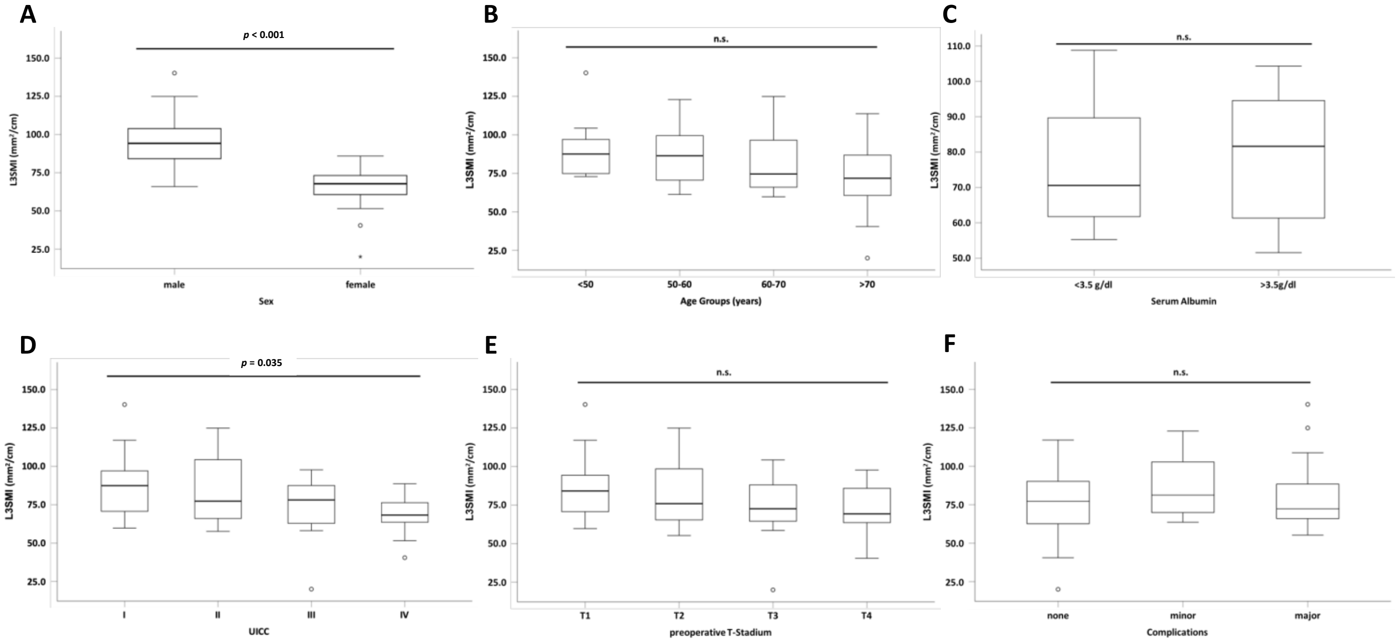

Figure S3. Representation of Figure 2 in mm²/cm.

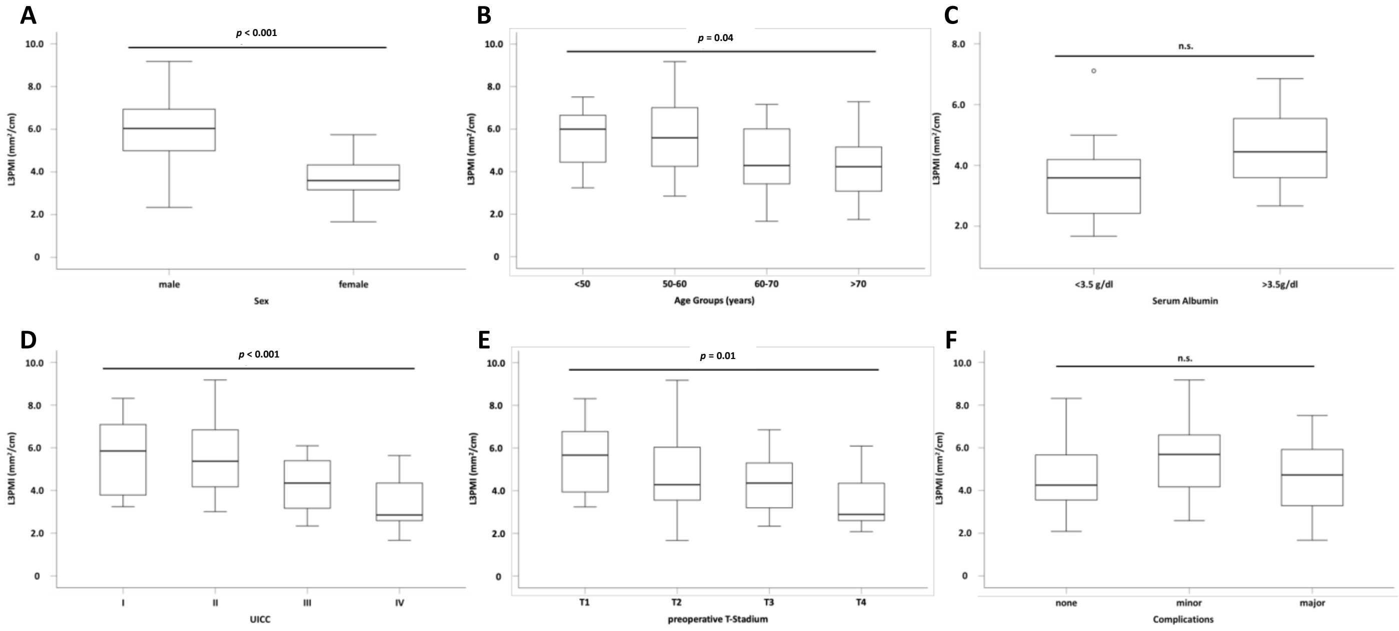

Figure S4. Representation of Figure 3 in mm²/cm.

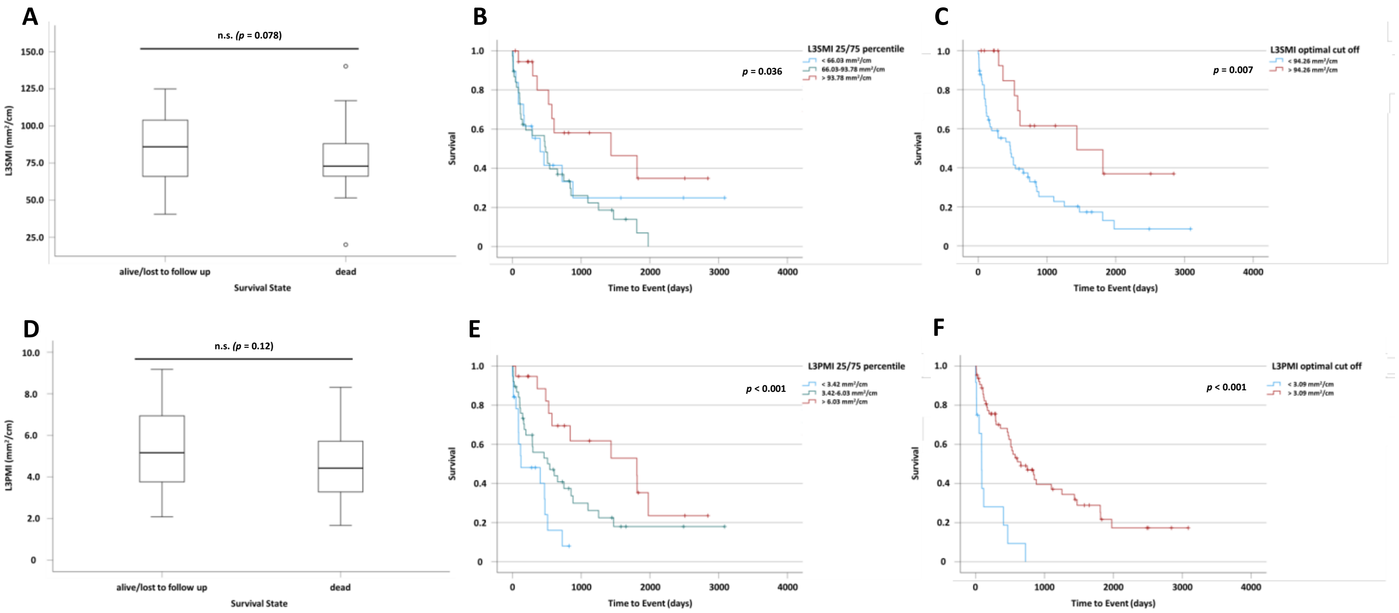

Figure S5. Representation of Figure 4 in mm²/cm.

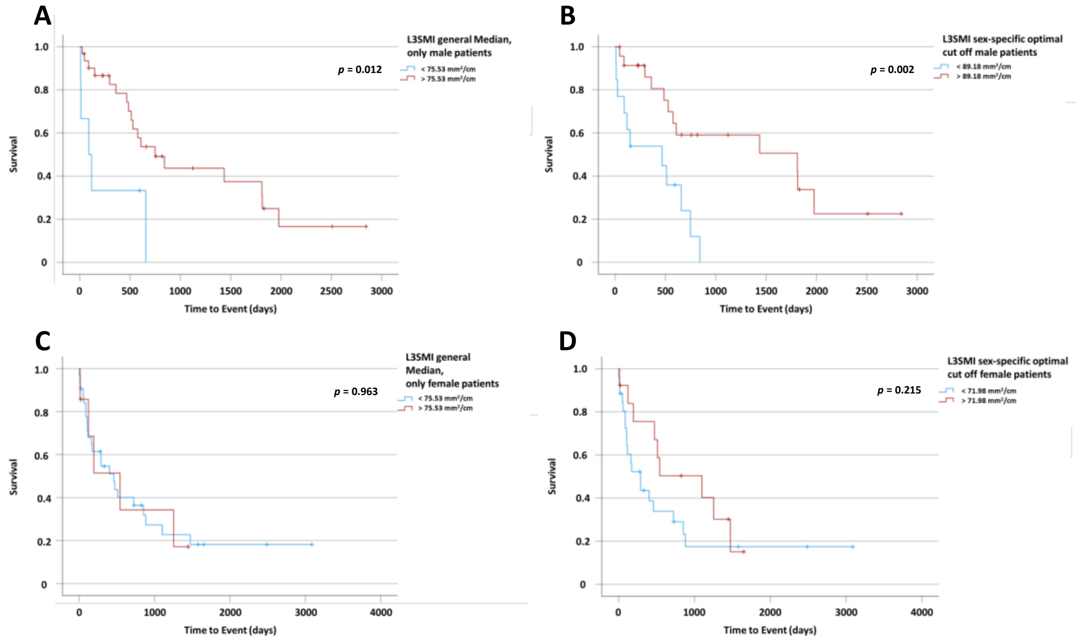

Figure S6. Representation of Figure 5 in mm²/cm.

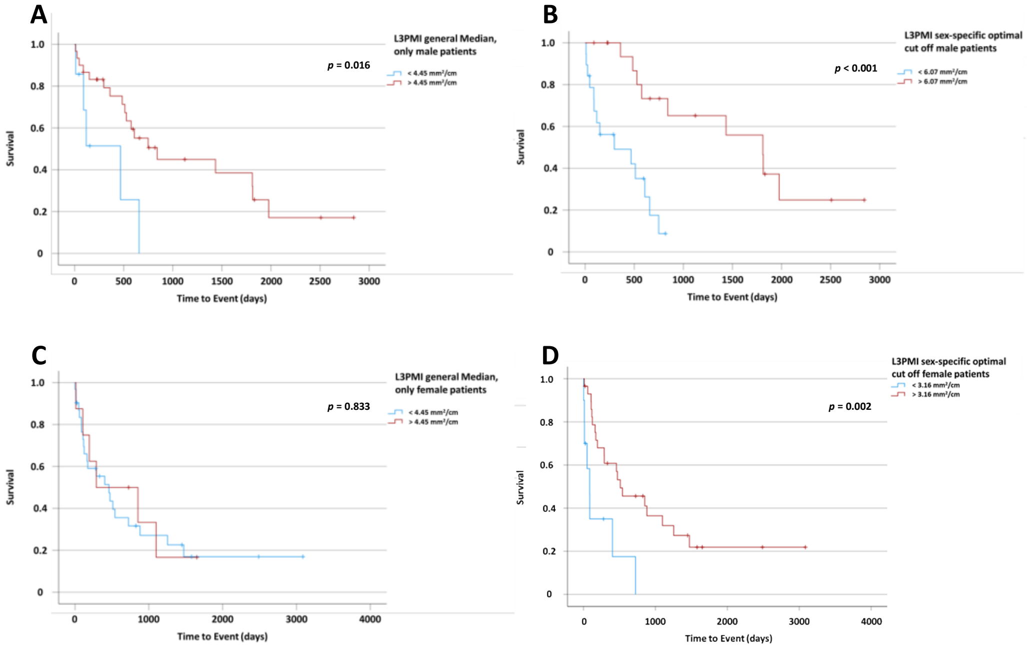

Figure S7. Representation of Figure 6 in mm²/cm.

Table S1. ROC-Analysis of L3SMI, L3PMI, UICC stage and age.

	AUC (survival)			
	**3-month**	**6-month**	**12-month**	**Overall**
**UICC-stage**	0.72	0.75	0.71	0.62
**Age**	0.68	0.72	0.68	0.62
**L3SMI (cm²/m²)**	0.62	0.65	0.61	0.64
**L3PMI (cm²/m²)**	0.73	0.73	0.65	0.62
